# Efficacy and safety of dexamethasone or triamcinolone in combination with anti-vascular endothelial growth factor therapy for diabetic macular edema: A systematic review and meta-analysis with trial sequential analysis

**DOI:** 10.1371/journal.pone.0318373

**Published:** 2025-02-07

**Authors:** Bo Zhou, Hua Liu, Feng Xiong

**Affiliations:** Chengdu Aidi Eye Hospital, Chengdu, China; Cairo University Kasr Alainy Faculty of Medicine, EGYPT

## Abstract

**Background:**

The clinical efficacy of anti-vascular endothelial growth factors (anti-VEGFs), corticosteroids, and their combined treatment for diabetic macular edema (DME) has been substantiated by numerous studies. However, it remains uncertain whether the therapeutic benefits of the combined treatment with corticosteroids and anti-VEGFs is superior to those of anti-VEGF monotherapy. Consequently, we conducted a meta-analysis to compare the efficacy and safety of combined treatment with dexamethasone or triamcinolone and anti-VEGF versus anti-VEGF monotherapy in DME treatment.

**Methods:**

An exhaustive search of the literature was performed on February 23, 2024, scanning through the databases including PubMed, Web of Science, Embase, and the Cochrane Library, with the aim of identifying all relevant studies. The combined results for efficacy and safety were analyzed using the standard mean difference (SMD) and relative risk (RR), both of which were presented with 95% confidence interval (CI). The assessment of heterogeneity was conducted via Cochran’s Q test, I^2^ statistics, and the implementation of a 95% prediction interval (PI). All analyses were performed by R 4.3.1, Stata 12.0, and TSA v0.9.5.10 Beta software.

**Results:**

This meta-analysis incorporated 21 eligible studies. The overall analysis revealed that combined treatment of dexamethasone or triamcinolone with anti-VEGF agents did not demonstrate superiority over anti-VEGF monotherapy in improving best-corrected visual acuity (BCVA) (Dexamethasone: SMD -0.266, 95% CI -1.001 to 0.468, 95% PI -2.878 to 2.346; Triamcinolone: SMD -0.340, 95% CI -1.230 to 0.550, 95% PI -3.554 to 2.874) and reducing central macular thickness (CMT) (Dexamethasone: SMD -1.255, 95% CI -2.861 to 0.350; Triamcinolone: SMD -0.207, 95% CI -0.895 to 0.481, 95% PI -2.629 to 2.215). However, the combination therapy significantly increased the risk of elevated intraocular pressure (RR 5.783, 95% CI 3.007 to 11.121, 95% PI 0.520 to 56.931) and ocular hypertension (RR 8.885, 95% CI 2.756 to 28.649, 95% PI 1.262 to 39.208). Subgroup analysis suggests that dexamethasone plus anti-VEGF therapy showed a greater reduction in central subfield thickness (SMD -0.440, 95% CI -0.755 to -0.126) compared to anti-VEGF monotherapy among patients with persistent DME.

**Conclusion:**

Our study confirmed that dexamethasone or triamcinolone plus anti-VEGF therapy did not show superior efficacy in improving BCVA and reducing CMT in DME patients compared with anti-VEGF monotherapy. Clinicians should weigh the pros and cons comprehensively when implementing combined therapy.

## 1. Introduction

Diabetes, a persistent metabolic disorder, precipitates an array of both microvascular and macrovascular complications. Global estimates predict a surge in diabetes mellitus diagnoses, potentially reaching 700 million by 2045 [1]. As the global count of adults grappling with diabetes and clinically significant macular oedema is expected to swell from roughly 18.83 million in 2020 to 28.61 million by 2045, the importance of streamlined training and care delivery becomes increasingly significant [2]. A common complication of diabetes mellitus, diabetic macular edema (DME), is characterized by abnormal fluid accumulation in the macula [3]. If not addressed, DME can trigger progressive retinal dysfunction, leading to an irreversible and permanent vision loss [4]. The arsenal of approved DME treatments encompasses intravitreal anti-vascular endothelial growth factor (anti-VEGF) agents, laser photocoagulation, and steroids [5]. However, due to its limited impact on visual enhancement, laser photocoagulation typically serves as a rescue therapy for DME. The advent of intravitreal injections of anti-VEGF agents has revolutionised DME treatment, rapidly emerging as the first-line treatment due to their dual ability to enhance visual and anatomical outcomes and avoid laser-associated complications such as subretinal fibrosis and laser scars [6]. Approved anti-VEGF treatments for DME management include aflibercept and ranibizumab, though the available options may vary by country, with off-label bevacizumab being utilised in certain regions [7].

Corticosteroids represent an alternative therapeutic strategy for DME due to their ability to diminish VEGFs and inflammatory markers, mitigate leukostasis, and enhance the integrity of vascular endothelial cell tight junctions [8]. Triamcinolone, an ocular steroid, exhibits anti-inflammatory and anti-angiogenic properties, and has been identified as a cost-effective option in DME management through multiple studies [9]. In regions with lower to middle economic status, intravitreal triamcinolone could serve as a viable, cost-effective substitute to anti-VEGF agents, provided the potential side effects such as cataract formation and increased intraocular pressure (IOP) are less significant than the risks of non-adherence due to the high cost of anti-VEGF treatments [10]. Within the array of corticosteroids deployed in eye care, dexamethasone exhibits superior clinical efficacy. The dexamethasone implant (Ozurdex), a biodegradable device engineered for sustained dexamethasone delivery over an approximate six-month period, has garnered approval for the management of DME [11].

A plethora of research has substantiated the significant improvement in visual and anatomical outcomes in DME treatment, whether utilizing anti-VEGF agents or corticosteroids as monotherapies, or their combined application [5, 12, 13]. Nevertheless, it remains uncertain whether the integration of corticosteroids and anti-VEGF agents offers superior clinical advantages compared with the exclusive use of anti-VEGF agents. A preceding meta-analysis by Namvar et al. evaluated the efficacy and safety of intravitreal anti-VEGF monotherapy versus its combination with corticosteroids in treating macular edema secondary to retinal vein occlusion [14]. In this context, we performed a comprehensive meta-analysis aiming to systematically compare the efficacy and safety of corticosteroids (primarily dexamethasone or triamcinolone) combined with anti-VEGF therapy versus anti-VEGF monotherapy in the management of DME.

## 2. Methods

This meta-analysis was undertaken in compliance with the Preferred Reporting Items for Systematic Reviews and Meta-Analyses (PRISMA) guidelines (S1 Checklist) [15]. Furthermore, the study protocol was duly registered in the PROSPERO database under the identifier CRD42024517791.

### 2.1 Search strategy

An exhaustive search of electronic databases, including PubMed, Web of Science, Embase, and the Cochrane Library, was conducted to identify pertinent studies published in English up to February 23, 2024. The key words for the literature search included: (“glucocorticoid” OR “glucocorticoids” OR “corticosteroid” OR “corticosteroids” OR “adrenal cortex hormone” OR “dexamethasone” OR “dexasone” OR “methylfluorprednisolone” OR “triamcinolone”) AND (“anti-VEGF” OR “anti-vascular endothelial growth factor” OR “ranibizumab” OR “aflibercept” OR “bevacizumab”) AND (“macular edema” OR “Irvine-Gass syndrome” OR “cystoid macular dystrophy”). S1 File provided a detailed description of the search strategies employed. Moreover, to ensure completeness of the study capture, we scrutinized the reference lists of eligible studies and associated review articles.

### 2.2 Inclusion and exclusion criteria

The criteria for the selection of eligible studies included: (1) cohort studies, or randomized controlled trials (RCTs); (2) combination therapy group: dexamethasone or triamcinolone combined with anti-VEGF (e.g., bevacizumab, ranibizumab, or aflibercept) treatment; (3) control group: anti-VEGF monotherapy; (4) studies reporting at least one of the following outcomes: changes in best-corrected visual acuity (BCVA) in Early Treatment Diabetic Retinopathy Study (ETDRS) letters or logarithmic minimum angle of resolution (logMAR) values, central subfield thickness (CST), central macular thickness (CMT) or intraocular pressure (IOP) from baseline, and ocular adverse events (OAEs). The exclusion criteria were as follows: (1) non-comparative studies; (2) the research subjects were patients with macular edema secondary to retinal vein occlusion or other causes; (3) studies with duplicate original data; (4) reviews, case reports, conference abstracts, commentaries, and letters.

### 2.3 Data extraction

Data from the included studies were independently extracted by two reviewers and collated into a predefined data extraction table. This extraction encompassed elements such as the first author and publication year, study design, research location, type of DME, combination therapy and monotherapy strategies, age and sample size of DME patients, the count of eyes undergoing treatment, follow-up duration, and outcomes. The primary outcomes were changes in BCVA, CMT, and CST from baseline, while secondary outcomes were changes in IOP from baseline and the occurrence of OAEs. The increase in IOP was defined as an elevation of more than 5 mmHg compared to the baseline assessment [16], whereas ocular hypertension is defined as an IOP greater than 21 mmHg [17]. Given the heterogeneity in BCVA reporting, with some studies using ETDRS letters and others employing logMAR visual acuity, all data were harmonized into logMAR visual acuity for a robust analysis [18]. Any discrepancies that arose during this process were settled through discussions involving a third investigator.

### 2.4 Risk of bias assessment

The assessment of bias risk was jointly undertaken by two reviewers, with any discord resolved by a third reviewer. The modified Jadad scale was utilized to determine the quality of RCTs, taking into account factors such as randomization, concealment of randomization, double-blinding, and tracking of withdrawals and dropouts. Studies were deemed to be of low quality if they scored between 0–3, and of high quality if they scored between 4–7. For the included cohort studies, quality was evaluated using the Newcastle-Ottawa Scale (NOS) [19], which considers subject selection, comparability of the exposure and control groups, and outcome assessment. Each cohort study was consequently deemed to be of low (0–3), moderate (4–6), or high (7–9) quality.

### 2.5 Statistical analysis

The standard mean difference (SMD) was employed to amalgamate continuous outcome data, and the findings were illustrated within a 95% confidence interval (CI). We harnessed the relative risk (RR) and 95% CI for dichotomous outcomes. The Cochran’s Q test, I^2^ statistics, and 95% prediction interval (PI) were used for heterogeneity evaluation. Acceptable heterogeneity was indicated by results with I^2^ < 50% or P > 0.10, leading to the application of a fixed-effects model; otherwise, a random-effects model was engaged [20]. Subgroup analysis was performed based on specific anti-VEGF drugs or DME types. Sensitivity analyses were conducted to verify the robustness of the current analysis. The potential presence of publication bias was evaluated through the visual inspection of funnel plots and the application of Begg’s and Egger’s tests. The trim-and-fill method was used for quantitative adjustment if publication bias was detected [21]. All analyses were performed using R 4.3.1 and Stata 12.0 (Stata Corp. College Station, Texas, USA) software, with statistical significance defined by a two-tailed *p* < 0.05.

### 2.6 Trial sequential analysis

To robustly assess the efficacy and safety of combining dexamethasone or triamcinolone with anti-VEGF agents in DME patients, we used trial sequential analysis (TSA) to ensure evidence strength and adjust for potential errors [22]. The TSA v0.9.5.10 Beta software (www.ctu.dk/tsa) was utilized to carry out TSA, which aided in ascertaining the trial sequential monitoring boundary and in setting the required information size (RIS) boundary. O’Brien-Fleming α-spending boundaries were established in our study, with a stipulated type I error rate of 5% and a statistical power of 80%, both parameters being two-tailed. When the cumulative Z-curve intersected with either the trial sequential monitoring boundary or the RIS boundary, further exploration was considered superfluous. This intersection point provided conclusive proof to either endorse or refute the impact of the intervention.

## 3. Results

### 3.1 Study selection

**Fig 1:**
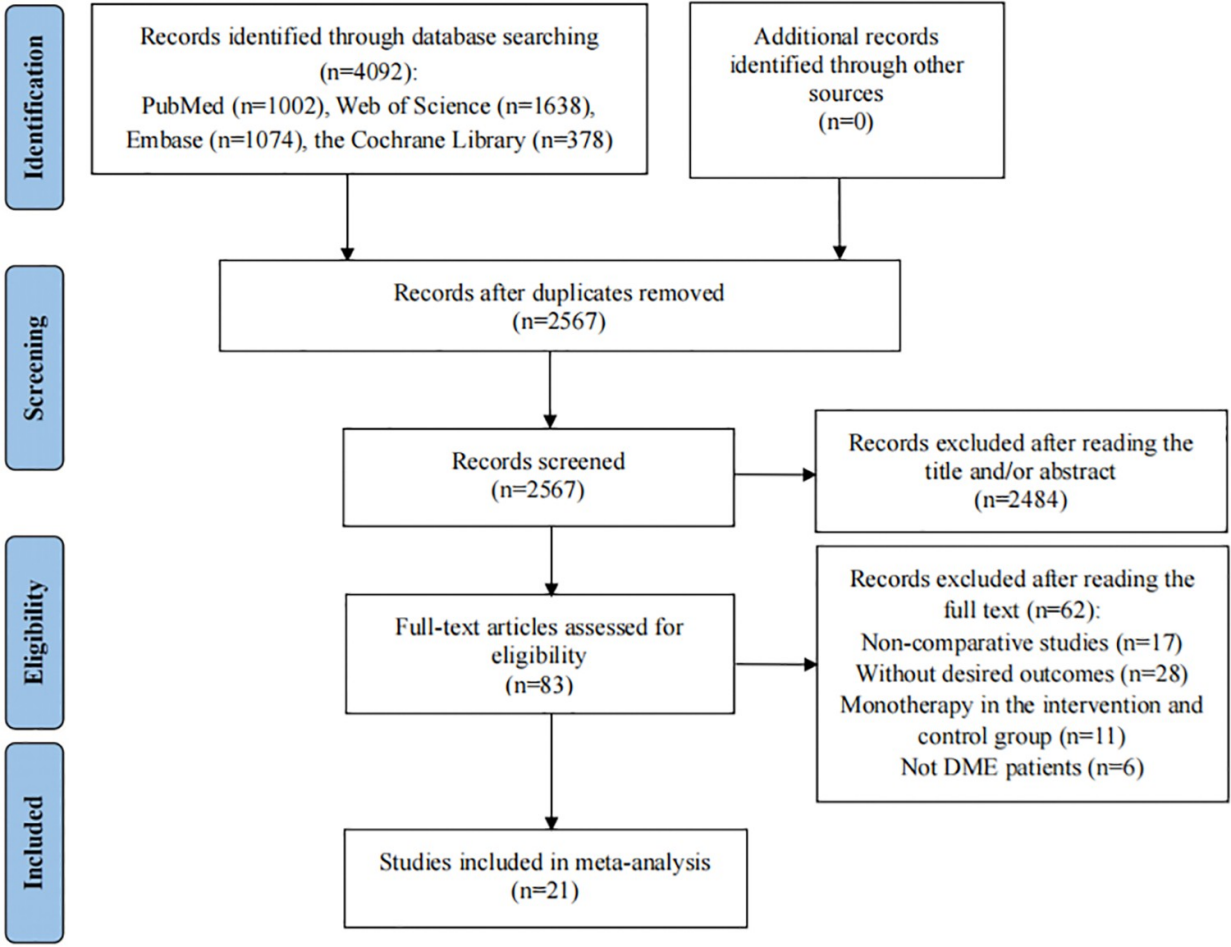
Flow diagram of the process of study selection.

The preliminary search in PubMed, Web of Science, Embase, and the Cochrane Library yielded 4,092 entries. Following the exclusion of 1,525 duplicate entries, we were left with 2,567 unique records. Subsequently, a screening of titles and abstracts was carried out on these 2,567 papers, during which 2,484 papers were removed due to their lack of relevance to our study (S1 Data). A thorough full-text review of the remaining 83 articles led to the dismissal of 62 articles based on our inclusion/exclusion criteria: 17 were non-comparative research; 28 did not offer the required efficacy or safety outcomes; 11 had both intervention and control groups treated with monotherapy; and 6 did not focus on DME patients. Ultimately, our meta-analysis incorporated 21 studies [16, 17, 23–41] (Fig 1).

### 3.2 Study characteristics and quality assessment

Table 1 presented the characteristics of the studies and the participants involved in the research. The studies incorporated into our analysis comprised 14 RCTs and 7 cohort studies. These eligible studies, published in the span of 2008 to 2024, were conducted across Europe, Asia, North America, and Africa. Various types of DME were considered, including persistent, naive, refractory, center-involving, and resistant DME. DME remains unresolved or inadequately responsive after intravitreal anti-VEGF injections with at least 6 monthly intervals were defined as persistent DME [29]. Treatment methods for the combination group encompassed intravitreal injection of dexamethasone in conjunction with anti-VEGF agents, and intravitreal, suprachoroidal, or posterior subtenon injection of triamcinolone plus anti-VEGF drugs. The anti-VEGF medications used were bevacizumab, ranibizumab, and aflibercept. A total of 714 eyes were included in the combination group, while the monotherapy group comprised 722 eyes. The follow-up duration varied from 1 to 12 months. The overall scores for 10 RCTs and 5 cohort studies were between 4 to 7 and 7 to 9, respectively, suggesting a low risk of bias. 4 RCTs and 2 cohort studies were deemed of low quality due to insufficient detail in their study design (S2 File).

**Table 1:** Characteristics of included studies.

Study ID	Study design	Location	Disease	Treatment	Sample size (M/F)	Age (mean ± SD, years)	Number of eyes	Follow-up duration	Outcomes
Lin (2022)	RCS	China	DME	Intravitreal dexamethasone + aflibercept	20/21	65.1 ± 8.7	52	6 months	1
				Intravitreal aflibercept	26/14	63.4 ± 13.9	50		
Maturi (2018)	RCT	USA	Persistent DME	Intravitreal dexamethasone + ranibizumab	34/31	Median (IQR): 64 (59–69)	65	24 weeks	1, 2, 4
				Intravitreal ranibizumab	28/36	Median (IQR): 66 (59–71)	64		
Kaya (2021)	RCT	Turkey	Naive DME	Intravitreal dexamethasone + ranibizumab	12/12	64.6 ± 10.5	34	12 months	1
				Intravitreal ranibizumab	12/10	66.2 ± 8.8	34		
Ozsaygılı (2024)	RCS	Turkey	DME	Intravitreal dexamethasone + aflibercept	17/22	54.8 ± 06	39	12 months	4
				Intravitreal aflibercept	21/22	56.2 ± 2.1	43		
Maturi (2015)	RCT	USA	Persistent DME	Intravitreal dexamethasone + bevacizumab	13/17	61 ± 10	21	12 months	1, 2, 4
				Intravitreal bevacizumab			19		
Kaya (2023)	RCT	Turkey	Naive DME	Intravitreal dexamethasone + ranibizumab	12/12	64.6 ± 10.5	34	24 months	4
				Intravitreal ranibizumab	12/10	66.2 ± 8.8	34		
Hernández-Bel (2019)	RCS	Spain	Naive DME	Intravitreal dexamethasone + aflibercept	15	66.2 (mean age)	15	12 months	1, 3, 4
				Intravitreal aflibercept	15	69.4 (mean age)	15		
Limon (2021)	PCS	Turkey	Persistent DME	Intravitreal dexamethasone + bevacizumab	12/17	64.34 ± 8.7	35	3 months	4
				Intravitreal bevacizumab	14/16	63.20 ± 6.4	30		
Karimi (2023)	PCS	Iran	Refractory DME	Intravitreal dexamethasone + bevacizumab	9/19	62.66 ± 8.55	40	1 month	1, 3
				Intravitreal bevacizumab	14/19	62.73 ± 7.11	41		
Riazi-Esfahani (2018)	RCT	Iran	DME	Intravitreal triamcinolone + bevacizumab	21/25	62 ± 8.6	46	24 weeks	1, 3
				Intravitreal bevacizumab			46		
Soheilian (2012)	RCT	Iran	Naive DME	Intravitreal triamcinolone + bevacizumab	NA	60.9 ± 5.6	36	24 months	1, 3, 6
				Intravitreal bevacizumab			39		
Ahmadieh (2008)	RCT	Iran	Refractory DME	Intravitreal triamcinolone + bevacizumab	50/51	59.7 ± 8.3	37	24 weeks	1, 3, 6
				Intravitreal bevacizumab			41		
Yaseri (2014)	RCT	Iran	DME	Intravitreal triamcinolone + bevacizumab	11	NA	11	24 months	1, 3
				Intravitreal bevacizumab	8		8		
Fazel (2023)	RCT	Iran	Center-involving DME	Suprachoroidal triamcinolone + intravitreal bevacizumab	10/16	62.4 ± 6.2	26	12 weeks	1, 5
				Intravitreal bevacizumab	9/23	62.8 ± 5.8	32		
Lim (2012)	RCT	Korea	DME	Intravitreal triamcinolone + bevacizumab	16/18	58.4 ± 5.9	36	12 months	6
				Intravitreal bevacizumab	19/19	61.4 ± 6.7	38		
Shoeibi (2013)	RCT	Iran	Refractory DME	Intravitreal triamcinolone + bevacizumab	7/9	59.1 ± 8.1	37	13.3 ± 3.4 (months)	1, 3
				Intravitreal bevacizumab	7/8	60.4 ± 9.3	41		
Shahid (2022)	RCS	Pakistan	Resistant DME	Suprachoroidal triamcinolone + intravitreal bevacizumab	26/14	58.46 ± 3.62	20	1 month	3
				Intravitreal bevacizumab			20		
Faghihi (2008)	RCT	Iran	DME	Intravitreal triamcinolone + bevacizumab	NA	56 ± 7	41	16 weeks	1, 3
				Intravitreal bevacizumab		59 ± 6	42		
Barakat (2021)	RCT	USA	DME	Suprachoroidal triamcinolone + intravitreal aflibercept	26/10	59.8 ± 10.16	36	24 weeks	5, 6
				Intravitreal aflibercept	24/11	59.2 ± 12.89	35		
Marey (2011)	RCT	Egypt	DME	Intravitreal triamcinolone + bevacizumab	19/11	57.66 ± 7.44	30	12 weeks	6
				Intravitreal bevacizumab	16/14	57.60 ± 7.30	30		
Chiu (2021)	RCS	China	Center-involving DME	Posterior subtenon triamcinolone + Intravitreal ranibizumab	10/13	59.6 ± 8.1	23	12 months	6
				Intravitreal ranibizumab	14/6	63.3 ± 8.6	20		

M, male; F, female; SD, standard deviation; I, Intervention group; C, control group; DME, diabetic macular edema; IQR, interquartile range; RCS, retrospective cohort study; RCT, randomized controlled trial; PCS, prospective cohort study; NA, not available; 1, best-corrected visual acuity; 2, central subfield thickness; 3, central macular thickness; 4, incidence of increased intraocular pressure; 5, intraocular pressure; 6, ocular hypertension incidence.

### 3.3 Meta-analysis of dexamethasone plus anti-VEGF therapy for DME

**Fig 2:**
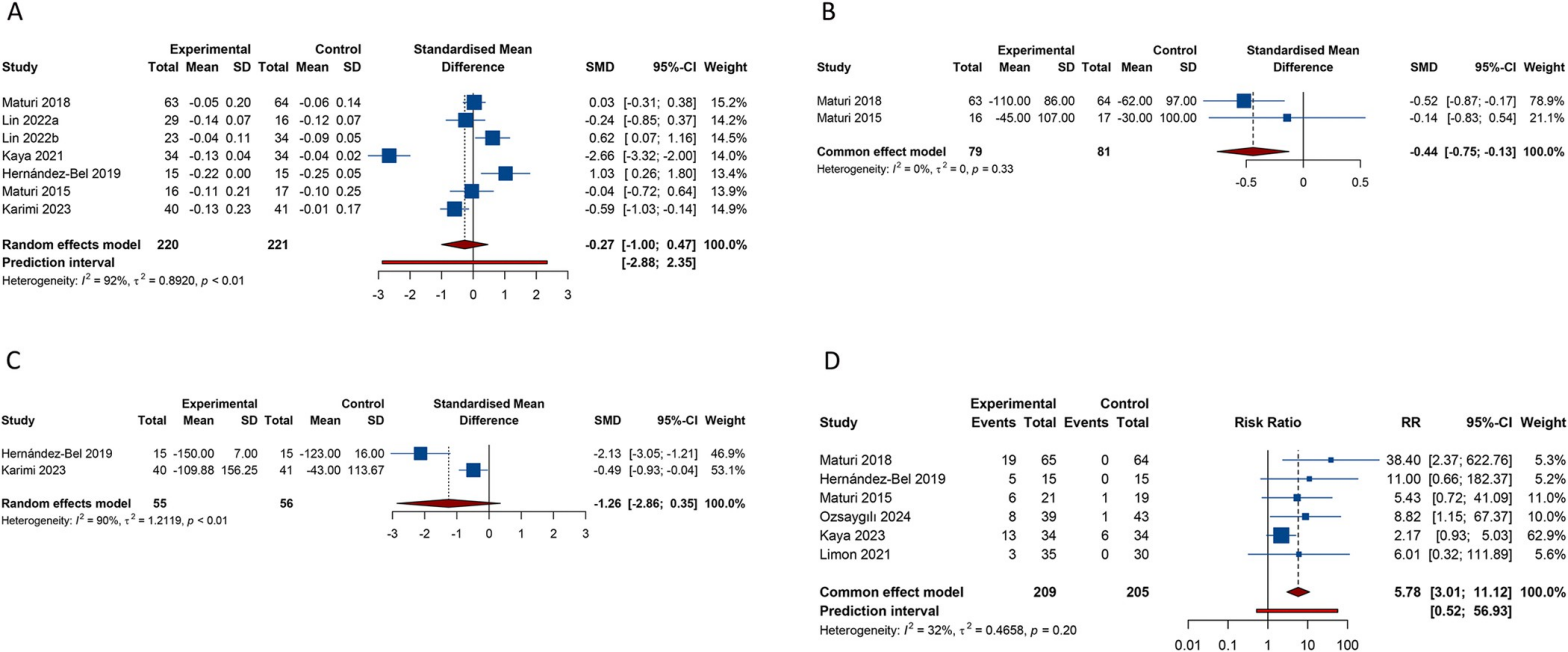
Forest plot of efficacy and safety outcomes after combination therapy of dexamethasone and anti-VEGF versus anti-VEGF monotherapy for DME. (A) Best-corrected visual acuity; (B) Central subfield thickness; (C) Central macular thickness; (D) Incidence of increased intraocular pressure.

A total of 7 studies employed BCVA as a metric for outcome measurement (S2 Data). Analysis using the random-effects model (I^2^ = 92.2%, Tau^2^ = 0.8920) disclosed that the improvement in BCVA following the combined application of dexamethasone and anti-VEGF agents did not significantly surpass the BCVA benefits of anti-VEGF monotherapy (SMD -0.266, 95% CI -1.001 to 0.468, 95% PI -2.878 to 2.346) (Table 2 and Fig 2A). Nevertheless, a more detailed subgroup analysis indicated that the combination of dexamethasone and bevacizumab resulted in greater BCVA improvement than using bevacizumab alone in patients with DME (SMD -0.426, 95% CI -0.799 to -0.053; I^2^ = 42.1%, Tau^2^ = 0.0629) (Table 3). The combination of dexamethasone with ranibizumab or aflibercept did not demonstrate a significant BCVA improvement compared to monotherapy with bevacizumab or aflibercept (all *p* >0.05) (Table 3). In cases of persistent or naive DME, our analysis found no significant difference in BCVA improvement between the combination of dexamethasone and anti-VEGF and anti-VEGF monotherapy (all *p* > 0.05) (Table 3). Similarly, regardless of whether the follow-up duration was less than or equal to 6 months or greater than 6 months, there was no significant difference in the improvement of BCVA between the combination therapy group and the monotherapy group (all *p* > 0.05) (Table 3).

**Table 2:** Pooled effect of the efficacy and safety of dexamethasone or triamcinolone combined with anti-VEGF therapy for diabetic macular edema.

Outcomes	Number of studies	Meta-analysis				Heterogeneity	
		SMD/RR	95% CI	*p* value	95% PI	I^2^, Tau^2^	P value
**Dexamethasone plus anti-VEGF vs. Anti-VEGF monotherapy**							
Best-corrected visual acuity	7	-0.266	-1.001, 0.468	0.477	-2.878, 2.346	92.2%, 0.8920	<0.001
Central subfield thickness	2	-0.440	-0.755, -0.126	**0.006**	-	0%, 0	0.335
Central macular thickness	2	-1.255	-2.861, 0.350	0.125	-	90.0%, 1.2119	0.002
Incidence of increased intraocular pressure	6	5.783	3.007, 11.121	**<0.001**	0.520, 56.931	32.1%, 0.4658	0.195
**Triamcinolone plus anti-VEGF vs. Anti-VEGF monotherapy**							
Best-corrected visual acuity	7	-0.340	-1.230, 0.550	0.454	-3.554, 2.874	95.0%, 1.3570	<0.001
Central macular thickness	7	-0.207	-0.895, 0.481	0.555	-2.629, 2.215	91.6%, 0.7644	<0.001
Intraocular pressure	2	0.270	-0.090, 0.629	0.141	-	0%, 0	0.943
Ocular hypertension incidence	6	8.885	2.756, 28.649	**<0.001**	1.262, 39.208	0%, 0	0.898

**Table 3:** Subgroup analysis of the efficacy and safety of dexamethasone or triamcinolone combined with anti-VEGF therapy for diabetic macular edema.

Outcomes and subgroups	Number of studies	Meta-analysis				Heterogeneity	
		SMD/RR	95% CI	*p* value	95% PI	I^2^, Tau^2^	P value
**Dexamethasone plus anti-VEGF vs. Anti-VEGF monotherapy**							
Best-corrected visual acuity							
Dexamethasone + Ranibizumab vs. Ranibizumab	2	-1.298	-3.939, 1.344	0.336	-	98.0%, 3.5605	<0.001
Dexamethasone + Aflibercept vs. Aflibercept	3	0.446	-0.259, 1.152	0.215	-7.705, 8.597	73.0%, 0.2820	0.025
Dexamethasone + Bevacizumab vs. Bevacizumab	2	-0.426	-0.799, -0.053	**0.025**	-	42.1%, 0.0629	0.189
Persistent DME	2	0.019	-0.291, 0.329	0.904	-	0%, 0	0.844
Naive DME	2	-0.822	-4.437, 2.794	0.656	-	98.0%, 6.6711	<0.001
Follow-up duration ≤ 6 months	4	-0.051	-0.526, 0.424	0.833	-1.584, 1.481	75.1%, 0.1732	0.007
Follow-up duration > 6 months	3	-0.564	-2.725, 1.597	0.609	-9.925, 8.796	96.5%, 3.5172	<0.001
Central subfield thickness							
Persistent DME	2	-0.440	-0.755, -0.126	**0.006**	-	0%, 0	0.335
Incidence of increased intraocular pressure							
Dexamethasone + Ranibizumab vs. Ranibizumab	2	7.423	0.231, 238.620	0.258	-	82.8%, 5.2990	0.016
Dexamethasone + Aflibercept vs. Aflibercept	2	9.571	1.843, 49.719	**0.007**	-	0%, 0	0.901
Dexamethasone + Bevacizumab vs. Bevacizumab	2	5.631	1.064, 29.805	**0.042**	-	0%, 0	0.955
Persistent DME	3	13.529	3.354, 54.573	**<0.001**	0.001, 97960.641	0%, 0	0.446
Follow-up duration ≤ 6 months	2	21.698	3.049, 154.387	**0.002**	-	0%, 0	0.342
Follow-up duration > 6 months	4	3.834	1.909, 7.697	**<0.001**	0.928, 11.268	2.0%, 0.0166	0.382
**Triamcinolone plus anti-VEGF vs. Anti-VEGF monotherapy**							
Best-corrected visual acuity							
Triamcinolone + Bevacizumab vs. Bevacizumab	7	-0.340	-1.230, 0.550	0.454	-3.554, 2.874	95.0%, 1.3570	<0.001
Refractory DME	2	-0.007	-3.290, 3.276	0.997	-	98.7%, 5.5417	<0.001
Follow-up duration ≤ 6 months	4	-1.002	-2.021, 0.018	0.054	-4.606, 2.603	94.0%, 1.0124	<0.001
Follow-up duration > 6 months	3	0.578	-0.609, 1.765	0.340	-4.436, 5.592	91.7%, 0.9913	<0.001
Central macular thickness							
Triamcinolone + Bevacizumab vs. Bevacizumab	7	-0.207	-0.895, 0.481	0.555	-2.629, 2.215	91.6%, 0.7644	<0.001
Refractory DME	2	0.461	-0.403, 1.325	0.296	-	86.1%, 0.3343	0.007
Follow-up duration ≤ 6 months	4	-0.850	-1.789, 0.089	0.076	-4.126, 2.427	92.6%, 0.8305	<0.001
Follow-up duration > 6 months	3	0.628	0.038, 1.218	**0.037**	-1.590, 2.846	67.3%, 0.1751	0.047
Intraocular pressure							
Follow-up duration ≤ 6 months	2	0.270	-0.090, 0.629	0.141	-2.059, 2.598	0%, 0	0.943
Ocular hypertension incidence							
Triamcinolone + Bevacizumab vs. Bevacizumab	4	11.200	2.687, 46.685	**0.001**	0.349, 223.314	0%, 0	0.756
Follow-up duration ≤ 6 months	3	4.491	0.785, 25.702	0.092	0.085, 209.117	0%, 0	0.875
Follow-up duration > 6 months	3	13.224	2.613, 66.932	**0.002**	0.288, 418.315	0%, 0	0.713

Two studies presented findings on CST and CMT, respectively. The amalgamated results from the fixed-effects model (I^2^ = 0%, Tau^2^ = 0) indicated that the degree of CST reduction was significantly greater in DME patients treated with dexamethasone combined with anti-VEGF drugs than in those treated with anti-VEGF alone (SMD -0.440, 95% CI -0.755 to -0.126) (Table 2 and Fig 2B), which was also observed in patients with persistent DME (Table 3). Regarding CMT, there was no significant difference in CMT reduction between dexamethasone plus anti-VEGF therapy and anti-VEGF monotherapy (SMD -1.255, 95% CI -2.861 to 0.350; I^2^ = 90.0%, Tau^2^ = 1.2119) (Table 2 and Fig 2C). Due to the limited number of studies included in the CMT analysis, subgroup analysis results with a minimum of 2 studies included have not yet been achieved.

With regard to OAEs, a total of 6 studies reported the outcome of increased IOP. The combined results from the fixed-effects model (I^2^ = 32.1%, Tau^2^ = 0.4658) suggested that the risk of increased IOP following the treatment of DME with the combination of dexamethasone and anti-VEGF was significantly higher than with anti-VEGF monotherapy (RR 5.783, 95% CI 3.007 to 11.121, 95% PI 0.520 to 56.931) (Table 2 and Fig 2D). Analysis grouped by the type of anti-VEGF agents showed that the incidence of increased IOP was significantly higher with the combination of dexamethasone and aflibercept or bevacizumab compared to aflibercept or bevacizumab monotherapy (Aflibercept: RR 9.571, 95% CI 1.843 to 49.719; I^2^ = 0%, Tau^2^ = 0; Bevacizumab: RR 5.631, 95% CI 1.064 to 29.805; I^2^ = 0%, Tau^2^ = 0) (Table 3). For persistent DME, the combined treatment of dexamethasone and anti-VEGF drugs revealed a significant increase in the risk of elevated IOP compared to the monotherapy of anti-VEGF (RR 13.529, 95% CI 3.354 to 54.573, 95% PI 0.001 to 97960.641; I^2^ = 0%, Tau^2^ = 0) (Table 3). Additionally, we observed that when the follow-up duration was either less than or equal to 6 months or greater than 6 months, the combination therapy of dexamethasone with anti-VEGF significantly increased the risk of elevated IOP compared to anti-VEGF monotherapy (all *p* < 0.05) (Table 3).

### 3.4 Meta-analysis of triamcinolone plus anti-VEGF therapy for DME

**Fig 3:**
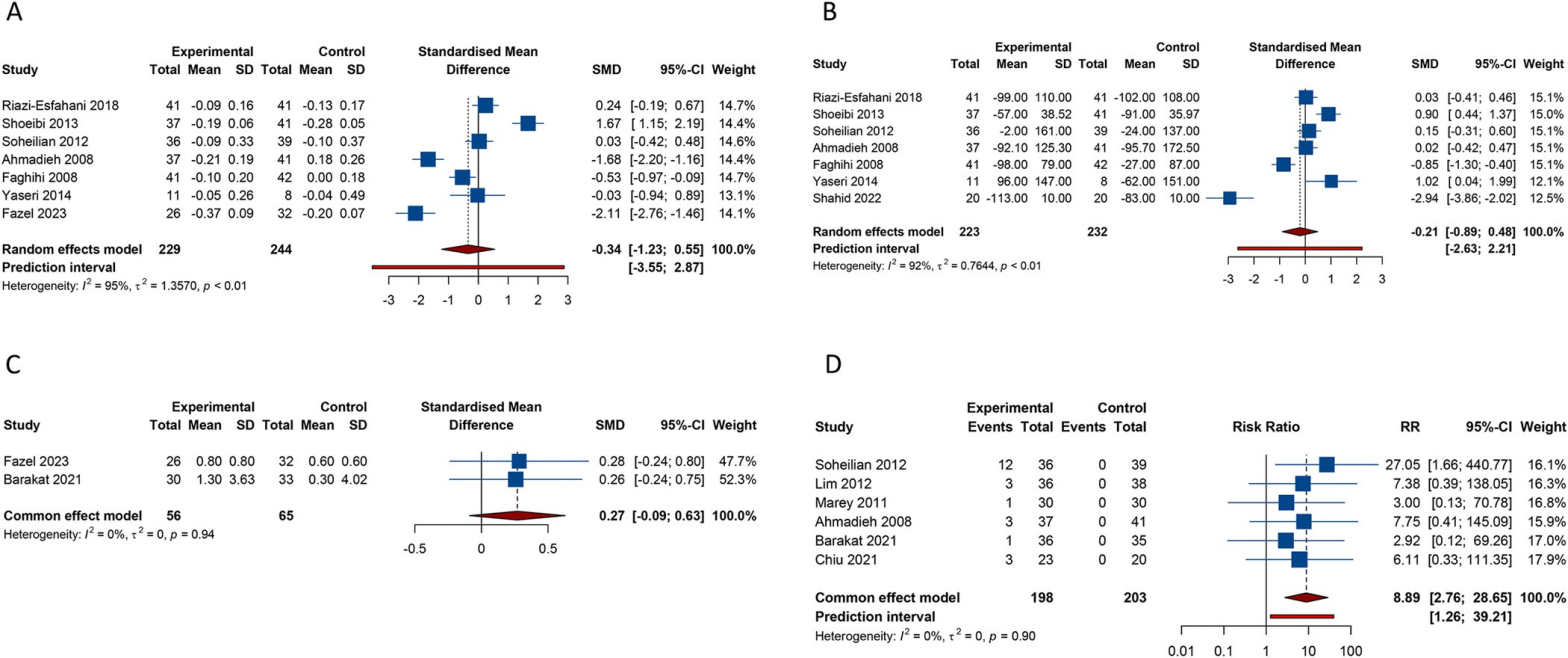
Forest plot of efficacy and safety outcomes after combination therapy of triamcinolone and anti-VEGF versus anti-VEGF monotherapy for DME. (A) Best-corrected visual acuity; (B) Central macular thickness; (C) Intraocular pressure; (D) Ocular hypertension incidence.

Seven studies reported BCVA as a primary outcome. Pooled results from the random-effects model (I^2^ = 95.0%, Tau^2^ = 1.3570) revealed that there was no noticeable difference in the improvement of BCVA between the group treated with the combination of triamcinolone and anti-VEGF agents and the group treated with anti-VEGF alone (SMD -0.340, 95% CI -1.230 to 0.550, 95% PI -3.554 to 2.874) (Table 2 and Fig 3A). A similar result for BCVA was observed when comparing the combined treatment of triamcinolone and bevacizumab with bevacizumab monotherapy (Table 3). Analysis grouped by DME type did not reveal a significant BCVA improvement in refractory DME patients receiving the combined treatment of triamcinolone and anti-VEGF drugs versus anti-VEGF monotherapy (SMD -0.007, 95% CI -3.290 to 3.276; I^2^ = 98.7%, Tau^2^ = 5.5417) (Table 3). Subgroup analyses stratified by duration of follow-up also revealed no significant findings in subgroups with follow-up times of ≤ 6 months and those greater than 6 months (all *p* > 0.05) (Table 3).

Seven studies reported on CMT. The consolidated findings from the random-effects model (I^2^ = 91.6%, Tau^2^ = 0.7644) indicated that the reduction in CMT following the combined therapy of triamcinolone and anti-VEGF agents was not significantly superior to that achieved with anti-VEGF monotherapy (SMD -0.207, 95% CI -0.895 to 0.481, 95% PI -2.629 to 2.215) (Table 2 and Fig 3B). A similar result for CMT was also found in the comparison between the combination treatment of triamcinolone and bevacizumab and bevacizumab monotherapy (Table 3). Subgroup analysis by DME type did not observe a significant CMT reduction in refractory DME patients with the combined treatment of triamcinolone and anti-VEGF drugs compared with anti-VEGF monotherapy (SMD 0.461, 95% CI -0.403 to 1.325; I^2^ = 86.1%, Tau^2^ = 0.3343) (Table 3). When the follow-up period was ≤ 6 months, the combination therapy of triamcinolone with anti-VEGF did not significantly improve the reduction of CMT compared to anti-VEGF monotherapy (SMD -0.850, 95% CI -1.789 to 0.089, 95% PI -4.126 to 2.427; I^2^ = 92.6%, Tau^2^ = 0.8305). However, for follow-up periods exceeding 6 months, we observed that the combination therapy was more effective in reducing CMT (SMD 0.628, 95% CI 0.038 to 1.218, 95% PI -1.590 to 2.846; I^2^ = 67.3%, Tau^2^ = 0.1751) (Table 3).

Two studies compared the increase in IOP after the combined treatment of triamcinolone and anti-VEGF versus the increase after monotherapy with anti-VEGF. The results revealed that there was no significant difference in the increase of IOP between the group treated with the combination of triamcinolone and anti-VEGF agents and the group treated with anti-VEGF alone (SMD 0.270, 95% CI -0.090 to 0.629; I^2^ = 0%, Tau^2^ = 0) (Table 2 and Fig 3C). Since the follow-up durations in both included studies were less than or equal to 6 months, the subgroup analyses stratified by follow-up time yielded consistent non-significant results (Table 3).

In terms of OAEs, a total of 6 studies reported the outcome of ocular hypertension. Pooled results from the fixed-effects model (I^2^ = 0%, Tau^2^ = 0) showed that the risk of ocular hypertension following the treatment of DME with the combination of triamcinolone and anti-VEGF was significantly higher than with the therapy of anti-VEGF alone (RR 8.885, 95% CI 2.756 to 28.649, 95% PI 1.262 to 39.208) (Table 2 and Fig 3D). When grouped by the type of anti-VEGF agents, the incidence of ocular hypertension caused by the combination treatment of triamcinolone and bevacizumab was significantly higher than that of bevacizumab monotherapy (RR 11.200, 95% CI 2.687 to 46.685, 95% PI 0.349 to 223.314; I^2^ = 0%, Tau^2^ = 0) (Table 3). Subgroup analysis based on follow-up duration indicated that, for follow-up periods exceeding 6 months, combination therapy significantly increased the risk of ocular hypertension compared to monotherapy (RR 13.224, 95% CI 2.613 to 66.932, 95% PI 0.288 to 418.315; I^2^ = 0%, Tau^2^ = 0). However, this significant difference was not observed in subgroup with follow-up durations ≤ six months (RR 4.491, 95% CI 0.785 to 25.702, 95% PI 0.085 to 209.117; I^2^ = 0%, Tau^2^ = 0) (Table 3).

### 3.5 Trial sequential analysis results

**Fig 4:**
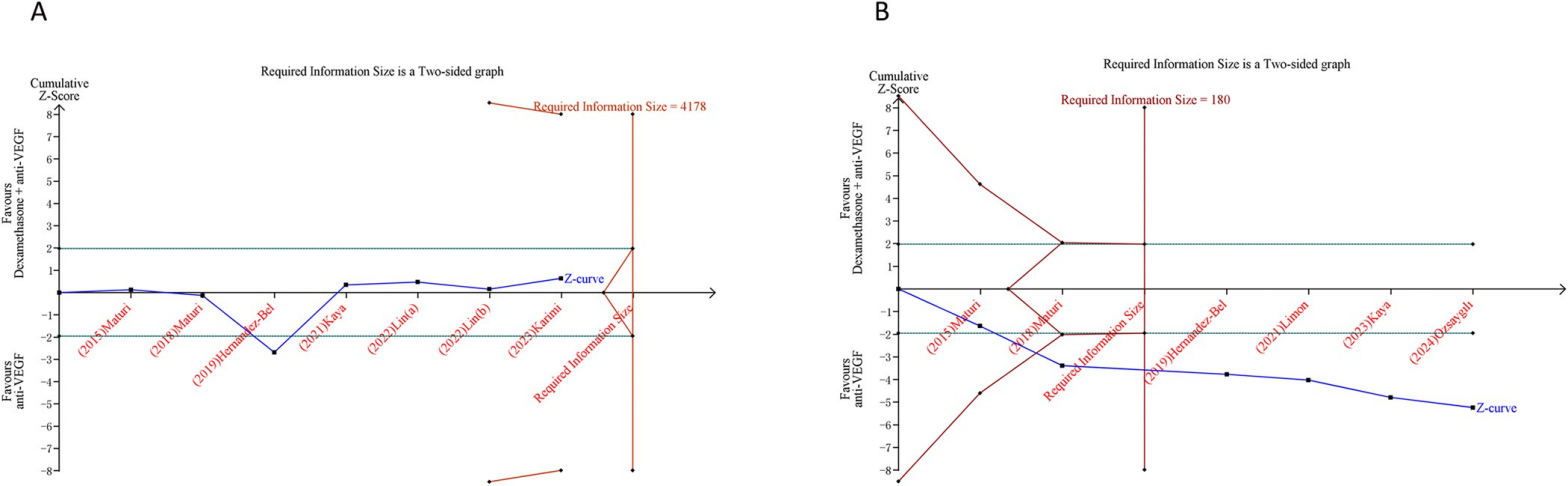
Trial sequential analysis of efficacy and safety outcomes after combination therapy of dexamethasone and anti-VEGF versus anti-VEGF monotherapy for DME. (A) Best-corrected visual acuity; (B) Incidence of increased intraocular pressure. Uppermost and lowermost red curves represent trial sequential monitoring boundary lines for benefit and harm, respectively. Inner red lines represent the futility boundary. Blue line represents evolution of cumulative Z-score. Horizontal green lines represent the conventional boundaries for statistical significance. Cumulative Z-curve crossing the trial sequential monitoring boundary or the RIS boundary provides firm evidence of effect.

**Fig 5:**
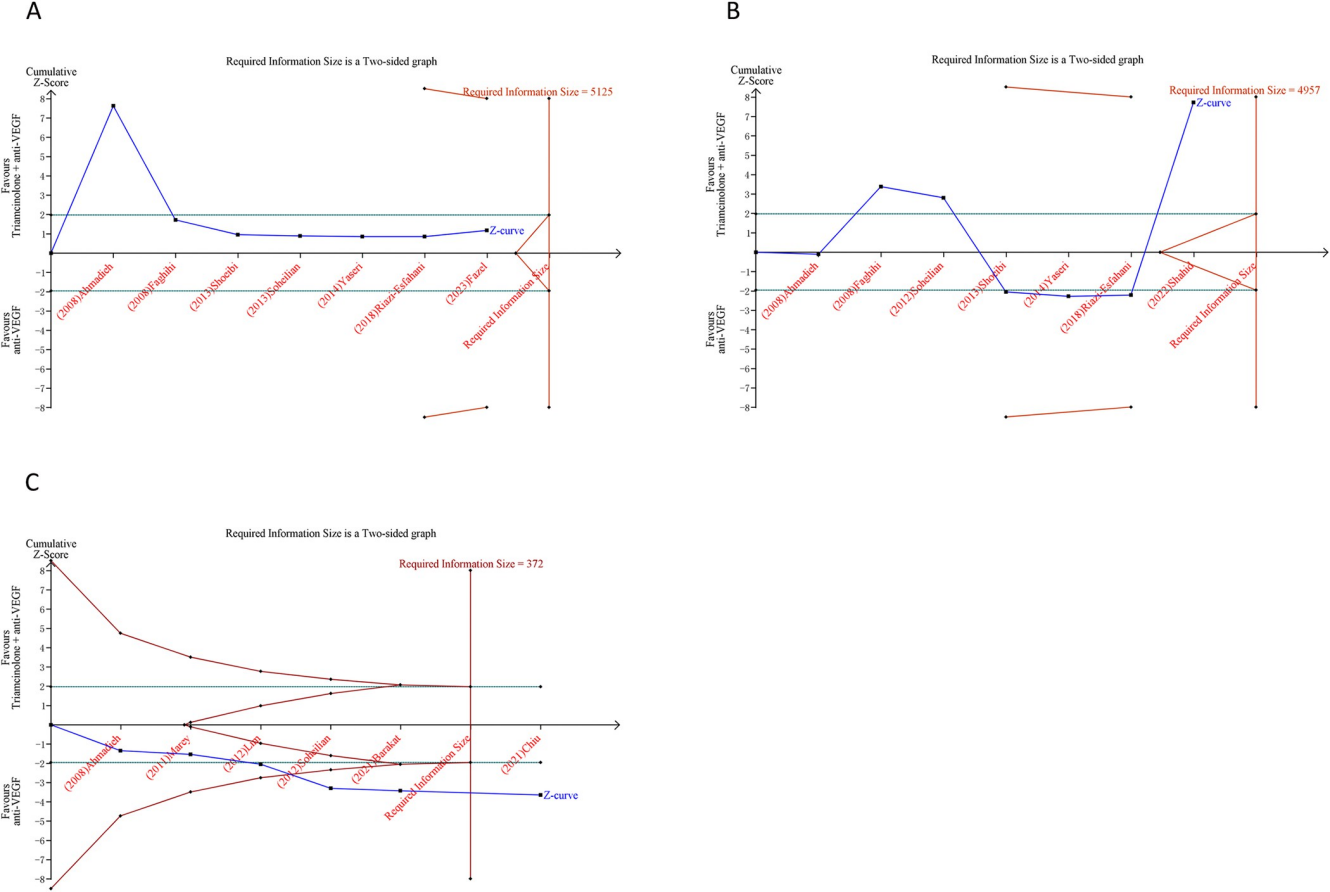
Trial sequential analysis of efficacy and safety outcomes after combination therapy of triamcinolone and anti-VEGF versus anti-VEGF monotherapy for DME. (A) Best-corrected visual acuity; (B) Central macular thickness; (C) Ocular hypertension incidence. Uppermost and lowermost red curves represent trial sequential monitoring boundary lines for benefit and harm, respectively. Inner red lines represent the futility boundary. Blue line represents evolution of cumulative Z-score. Horizontal green lines represent the conventional boundaries for statistical significance. Cumulative Z-curve crossing the trial sequential monitoring boundary or the RIS boundary provides firm evidence of effect.

In our research, we implemented TSA on outcomes that incorporated more than two studies. Regarding the outcomes following the combination therapy of dexamethasone and anti-VEGF, the cumulative Z-curve for BCVA failed to breach the trial sequential monitoring boundary or the RIS boundary. This implies a constrained capacity to draw a definitive conclusion concerning BCVA, potentially attributable to the existence of false positive. Conversely, the cumulative Z-curve corresponding to elevated IOP intersected both the trial sequential monitoring boundary and the RIS boundary, hinting that a fairly conclusive inference concerning the elevated IOP can be drawn (Fig 4). Similarly, in the context of outcomes after the combined treatment of triamcinolone and anti-VEGF, the cumulative Z-curves for BCVA and CMT did not intersect either the RIS boundary or the trial sequential monitoring boundary. In contrast, the cumulative Z-curve for ocular hypertension did cross both the RIS boundary and the trial sequential monitoring boundary (Fig 5).

### 3.6 Sensitivity analysis and publication bias

We performed sensitivity analysis and tested for publication bias on pooled results that included six or more studies. During the sensitivity analysis, we calculated the pooled SMDs and RRs and their associated 95% CIs, sequentially excluding individual studies to assess their potential impact on the comprehensive results. The sensitivity analysis suggested that the studies by Kaya in 2021 and 2023 might be contributing to the heterogeneity observed in BCVA and the incidence of increased IOP following the combined administration of dexamethasone and anti-VEGF, respectively. Additionally, the studies conducted by Fazel and Shahid were identified as potential sources of the pronounced heterogeneity observed in BCVA and CMT after triamcinolone plus anti-VEGF therapy, respectively. Results from both Begg’s and Egger’s tests indicated no significant publication bias across all the assessed efficacy and safety outcomes (all *p* > 0.05).

## 4. Discussion

A multitude of research has illustrated the advantages of using intravitreal anti-VEGF injections in treating DME, specifically in enhancing visual acuity and diminishing retinal thickening [42, 43]. Nevertheless, the need for repeated injections to maintain the effectiveness is a drawback of this treatment method. Consequently, recent DME treatments have explored additional or alternative approaches, including the use of intravitreal steroids in conjunction with anti-VEGF injections. This combination therapy, targeting different pathophysiological aspects through steroids and anti-VEGF agents, could potentially exhibit synergistic effects in DME treatment. However, there are no universally recognized guidelines for this combination therapy [44], and the evidence supporting the use of licensed intravitreal anti-VEGF injections with intravitreal steroid implants is limited [45]. Dexamethasone and triamcinolone, currently the most commonly used steroids in combination with anti-VEGF for the treatment of DME, have been reported in numerous studies for their effectiveness in conjunction with anti-VEGF for DME. Yet, it remains uncertain whether the combined therapy yields superior visual and anatomical results compared to anti-VEGF monotherapy. In this research, we systematically compared the clinical efficacy and safety of DME treatment with dexamethasone or triamcinolone in combination with anti-VEGF versus anti-VEGF monotherapy through a meta-analysis. Our findings suggested that dexamethasone or triamcinolone combined with anti-VEGF agents did not significantly improve BCVA nor did it reduce CMT compared with anti-VEGF monotherapy. However, further subgroup analysis confirmed that the combination therapy of dexamethasone and bevacizumab led to a more pronounced improvement in BCVA compared with bevacizumab monotherapy. Moreover, for patients with persistent DME, the combination therapy of dexamethasone and anti-VEGF showed a greater reduction in CST compared to anti-VEGF monotherapy.

Dexamethasone stands as the corticosteroid with the highest relative clinical efficacy in ophthalmological applications, exerting its multifaceted impacts through the modulation of numerous signal transduction pathways. A meta-analysis conducted in 2018 compared dexamethasone therapy with anti-VEGF therapy, incorporating only RCTs from a total of four studies [46]. The pooled findings suggested comparable effects between dexamethasone and anti-VEGF therapy on visual acuity enhancement, with the former demonstrating superior anatomical outcomes. These results align with the findings from our study. The potency of dexamethasone in managing persistent DME may be attributed to its robust anti-inflammatory, anti-edema, and anti-angiogenic properties. As previously discussed, corticosteroids have the capacity to reduce the expression of VEGF and proinflammatory cytokines, curtail vascular leakage, and inhibit leukostasis [47]. Two subsequent meta-analyses were undertaken more recently. Chi et al. incorporated 2,409 eyes into their study and found no noticeable disparities between dexamethasone implant and anti-VEGF in the improvement of BCVA from the combined analysis of non-resistant eyes [48]. In a similar vein, Patil et al.’s study incorporated 14 RCTs, encompassing a total of 827 eyes, and reported no significant difference between intravitreal steroids and anti-VEGF agents in BCVA improvement. Notably, a significant reduction in retinal thickness at 3 and 6 months and at the final follow-up was observed in the dexamethasone implant group [49]. In our study, the combination of dexamethasone and bevacizumab demonstrated a significant improvement in BCVA compared with bevacizumab monotherapy, while the combination of dexamethasone with ranibizumab or aflibercept did not show differential results in improving BCVA compared to ranibizumab or aflibercept monotherapy. These results, however, do not necessarily confirm that the combination of dexamethasone and bevacizumab would yield superior BCVA improvements in clinical practice. The limited number of included studies restricts the generalizability of our subgroup analysis results to a broader population. Hence, the inclusion of more studies is essential to further explore the visual and anatomical benefits offered by the combination of dexamethasone with various types of anti-VEGFs in treating different types of DME.

Owing to its accessibility, cost-effectiveness, and dose-dependent duration of action, intravitreal triamcinolone frequently serves as a therapeutic option for DME [50]. Intravitreal triamcinolone, with a potential clinical activity span of up to three months, can mitigate the infiltration of inflammatory vessels and modulate VEGF expression, consequently reducing macular edema and augmenting visual acuity [30]. Although our final analysis failed to substantiate a significant improvement in BCVA with the combination of triamcinolone and anti-VEGF compared with anti-VEGF monotherapy, it is undeniable that DME patients treated with the combination of triamcinolone and anti-VEGF consistently exhibited a downward trend in logMAR BCVA from baseline across all included studies. This validates the effectiveness of the combined therapy with triamcinolone and anti-VEGF in improving BCVA, albeit not significantly when compared to anti-VEGF monotherapy. The capacity of triamcinolone to incite lipocortins is the basis for its anti-inflammatory and analgesic properties. These proteins, as shown in studies, are capable of reducing leukocyte chemotaxis, controlling biosynthesis, and preventing the discharge of arachidonic acid from the phospholipid membrane, a critical precursor of potent inflammatory cell mediators including leukotrienes and prostaglandins [51]. Certain studies have indicated that triamcinolone notably suppresses the expression of leukotriene B4, TNF-α, interleukin 1β, and thromboxane B2 in a dose-responsive manner. Furthermore, triamcinolone appears to curtail the expression of matrix metalloproteinase and downregulate intercellular adhesion molecule 1 in choroidal endothelial cells [52]. By targeting abnormal cell proliferation and restoring blood-retinal barrier function, triamcinolone proves effective in managing edematous and proliferative diseases [53]. A retrospective analysis demonstrated that the application of triamcinolone via posterior subtenon injection for treating DME in eyes that had undergone vitrectomy led to a reduction in CMT and an increase in BCVA [54]. Another research team appraised the efficacy and safety of triamcinolone delivery into the suprachoroidal area via a custom-made needle for DME treatment following pars plana vitrectomy, observing an enhancement in visual acuity and a significant decrease in central foveal thickness [55]. However, our study confirmed that the combined therapy of triamcinolone and anti-VEGF agents, primarily bevacizumab, did not yield superior improvement in visual acuity, significant reduction in CMT, or greater decrease in IOP compared with anti-VEGF monotherapy. This suggests that clinicians should exercise caution when considering a combination therapy with triamcinolone and anti-VEGF drugs, particularly bevacizumab, in treating DME patients. The therapeutic effect achieved by this combination therapy may not be superior to that of anti-VEGF monotherapy, but may actually increase the cost of clinical treatment. On the other hand, the combination therapy could increase the risk of AEs.

Cataract development and intraocular pressure elevation represent the principal AEs linked to the utilization of dexamethasone and triamcinolone [56, 57]. The increased risk of related AEs has to some extent limited the clinical application of these two drugs. Our research corroborated that the combination therapy of dexamethasone or triamcinolone with anti-VEGF for DME increased the risk of elevated IOP and the incidence of ocular hypertension, respectively. Due to the inconsistency in AE reporting across the studies included in our analysis, and the lack of explicit enumeration of AEs in both combination therapy groups and monotherapy groups among certain studies, we have yet to conduct a pooled analysis for other AEs, such as endophthalmitis and retinal hemorrhage. Further supplementation of this analysis is warranted in the future.

Our study presents certain limitations. First, considerable heterogeneity was observed across the studies included for virtually all primary outcomes. A possible explanation for the high heterogeneity could be attributed to the impact of various clinical variables on study results. These variables encompass study design, DME types, dosage of the administered medication, follow-up duration, and the method of triamcinolone administration. Second, TSA results suggested that a larger sample size is needed in future analyses to further substantiate our current pooled results concerning BCVA and CMT. Third, the OAEs included in our meta-analysis were limited to elevated intraocular pressure and ocular hypertension. Additional events, such as cataracts, retinal hemorrhage, and endophthalmitis, warrant further investigation. Fourth, a portion of BCVA data (logMAR) was transformed from ETDRS letters with limitation in terms of nonstandard charts. Fifth, since the included studies did not clearly specify or report the time points (such as six months post-treatment) for outcome measures, future meta-analyses could focus on comparing efficacy and safety outcomes at different intervals.

## 5. Conclusions

In summary, our study suggested that the combined treatment of dexamethasone or triamcinolone with anti-VEGF agents did not demonstrate superior efficacy in improving BCVA or reducing CMT compared with anti-VEGF monotherapy in DME patients. On the contrary, the combination therapy of dexamethasone or triamcinolone and anti-VEGF drugs significantly increased the risk of elevated IOP and ocular hypertension. Although further subgroup analysis revealed that the combined therapy of dexamethasone and anti-VEGF yielded greater BCVA improvement among patients with persistent DME, this finding requires validation in subsequent studies.

## Supporting information

S1 Checklist PRISMA 2020 checklist. (PDF)

S1 File Search strategy. (DOCX)

S2 File Risk of bias and quality assessments for each study. (DOCX)

S1 Data Studies excluded from the analyses with the reasons for exclusion. (XLSX)

S2 Data Data extracted from the included studies for meta-analysis. (XLSX)
