# Supplementary material for: Efficacy and safety of dexamethasone or triamcinolone in combination with anti-vascular endothelial growth factor therapy for diabetic macular edema: A systematic review and meta-analysis with trial sequential analysis
Source: PLoS One. 2025 Feb 7;20(2):e0318373. doi: 10.1371/journal.pone.0318373 (PMC11805578; doi:10.1371/journal.pone.0318373)
Supplement: S1 File — (DOCX) [file pone.0318373.s002.DOCX]

**PubMed 1002**

#1 (glucocorticoid) OR (glucocorticoids) OR (corticosteroid) OR (corticosteroids) OR (adrenal cortex hormone) OR (dexamethasone) OR (dexasone) OR (methylfluorprednisolone) OR (triamcinolone)

#2 (anti-vascular endothelial growth factors) OR (anti-VEGF) OR (bevacizumab) OR (ranibizumab) OR (aflibercept)

#3 (macular edema) OR (Irvine-Gass syndrome) OR (cystoid macular dystrophy)

#4 #1 AND #2 AND #3

**Web of Science 1638**

#1 TS=((glucocorticoid) OR (glucocorticoids) OR (corticosteroid) OR (corticosteroids) OR (adrenal cortex hormone) OR (dexamethasone) OR (dexasone) OR (methylfluorprednisolone) OR (triamcinolone))

#2 TS=((anti-vascular endothelial growth factors) OR (anti-VEGF) OR (bevacizumab) OR (ranibizumab) OR (aflibercept))

#3 TS=((macular edema) OR (Irvine-Gass syndrome) OR (cystoid macular dystrophy))

#4 #1 AND #2 AND #3

**Embase 1074**

#1 glucocorticoid:ti,ab,kw OR glucocorticoids:ti,ab,kw OR corticosteroid:ti,ab,kw OR corticosteroids:ti,ab,kw OR 'adrenal cortex hormone':ti,ab,kw OR dexamethasone:ti,ab,kw OR dexasone:ti,ab,kw OR methylfluorprednisolone:ti,ab,kw OR triamcinolone:ti,ab,kw

#2 'anti-vascular endothelial growth factors':ti,ab,kw OR 'anti vegf':ti,ab,kw OR bevacizumab:ti,ab,kw OR ranibizumab:ti,ab,kw OR aflibercept:ti,ab,kw

#3 'macular edema':ti,ab,kw OR 'irvine-gass syndrome':ti,ab,kw OR 'cystoid macular dystrophy':ti,ab,kw

#4 #1 AND #2 AND #3

**The Cochrane Library 378**

#1 All Text=((glucocorticoid) OR (glucocorticoids) OR (corticosteroid) OR (corticosteroids) OR (adrenal cortex hormone) OR (dexamethasone) OR (dexasone) OR (methylfluorprednisolone) OR (triamcinolone))

#2 All Tex=((anti-vascular endothelial growth factors) OR (anti-VEGF) OR (bevacizumab) OR (ranibizumab) OR (aflibercept))

#3 All Tex=((macular edema) OR (Irvine-Gass syndrome) OR (cystoid macular dystrophy))

#4 #1 AND #2 AND #3
