# Supplementary material for: Efficacy and safety of dexamethasone or triamcinolone in combination with anti-vascular endothelial growth factor therapy for diabetic macular edema: A systematic review and meta-analysis with trial sequential analysis
Source: PLoS One. 2025 Feb 7;20(2):e0318373. doi: 10.1371/journal.pone.0318373 (PMC11805578; doi:10.1371/journal.pone.0318373)
Supplement: S2 File — (DOCX) [file pone.0318373.s003.DOCX]

| Table S1. Quality analysis of the included randomized controlled trials by modified Jadad scale. | | | | | | | |  |
| --- | --- | --- | --- | --- | --- | --- | --- | --- |
| Article | Published Year | Randomization | Randomization concealment | Double blind | Withdrawals and dropouts | Score | Study quality | |
| DOI: 10.1001/jamaophthalmol.2017.4914 | 2018 | 2 | 2 | 2 | 1 | 7 | High | |
| DOI: 10.1038/s41433-020-0949-2 | 2021 | 1 | 1 | 0 | 1 | 3 | Low | |
| DOI: 10.1097/iae.0000000000000533 | 2015 | 1 | 1 | 1 | 1 | 4 | High | |
| DOI: 10.1080/02713683.2023.2168013 | 2023 | 1 | 1 | 0 | 1 | 3 | Low | |
| DOI: 10.1007/s10792-017-0496-4 | 2018 | 2 | 2 | 0 | 1 | 5 | High | |
| PMID: 23943683 | 2013 | 2 | 2 | 0 | 1 | 5 | High | |
| DOI: 10.1097/IAE.0b013e31822f55de | 2012 | 2 | 2 | 0 | 1 | 5 | High | |
| DOI: 10.1159/000331935 | 2012 | 2 | 2 | 0 | 0 | 4 | High | |
| DOI: 10.2147/opth.S22103 | 2011 | 1 | 1 | 0 | 0 | 2 | Low | |
| DOI: 10.1007/s00417-007-0688-0 | 2008 | 2 | 2 | 2 | 0 | 6 | High | |
| DOI: 10.1177/112067210801800614 | 2008 | 1 | 1 | 0 | 0 | 2 | Low | |
| DOI: 10.4103/2008-322x.150818 | 2014 | 2 | 1 | 1 | 1 | 5 | High | |
| DOI: 10.1186/s12886-023-02790-y | 2023 | 2 | 2 | 1 | 1 | 6 | High | |
| DOI: 10.1016/j.oret.2020.08.007 | 2021 | 2 | 2 | 2 | 1 | 7 | High | |

| **Table S2.** The Newcastle-Ottawa quality assessment scale of the included cohort studies. | | | | | | | | | | | | | |
| --- | --- | --- | --- | --- | --- | --- | --- | --- | --- | --- | --- | --- | --- |
| Article | Published Year | Selection | | | |  | Comparability | |  | Assessment of outcome | | | Total score |
|  |  | Representativeness of exposure arm(s) | Selection of the comparative arm(s) | Origin of exposure source | Demonstration that outcome of interest was not present at start of study |  | Studies controlling the most important factors | Studies controlling the other main factors |  | Assessment of outcome with independency | Adequacy of follow-up length | Lost to follow-up acceptable |  |
| DOI: 10.1007/s00592-021-01824-5 | 2022 | 1 | 1 | 1 | 1 |  | 1 | 1 |  | 1 | 0 | 1 | 8 |
| DOI: 10.1159/000489345 | 2019 | 1 | 1 | 1 | 1 |  | 1 | 0 |  | 1 | 1 | 1 | 8 |
| DOI: 10.1007/s10792-024-02963-8 | 2024 | 1 | 1 | 1 | 1 |  | 1 | 1 |  | 1 | 1 | 1 | 9 |
| DOI: 10.1016/j.jfo.2020.08.033 | 2021 | 1 | 1 | 1 | 1 |  | 1 | 0 |  | 1 | 0 | 0 | 6 |
| DOI: 10.1016/j.jfo.2023.04.001 | 2023 | 1 | 1 | 1 | 1 |  | 1 | 1 |  | 1 | 0 | 0 | 7 |
| DOI: 10.53350/pjmhs22166301 | 2022 | 1 | 1 | 1 | 1 |  | 0 | 0 |  | 1 | 0 | 1 | 6 |
| DOI: 10.4103/tjo.tjo_31_20 | 2021 | 1 | 1 | 1 | 1 |  | 1 | 1 |  | 1 | 0 | 1 | 8 |
